# A Novel NADP-Dependent Formate Dehydrogenase From the Hyperthermophilic Archaeon *Thermococcus onnurineus* NA1

**DOI:** 10.3389/fmicb.2022.844735

**Published:** 2022-03-15

**Authors:** Ji-in Yang, Seong Hyuk Lee, Ji-Young Ryu, Hyun Sook Lee, Sung Gyun Kang

**Affiliations:** ^1^Marine Biotechnology Research Centre, Korea Institute of Ocean Science and Technology, Busan, South Korea; ^2^Department of Marine Biotechnology, KIOST School, University of Science and Technology, Daejeon, South Korea

**Keywords:** formate dehydrogenase, formate oxidation, NAD(P) reduction, ferredoxin reduction, carbon dioxide reduction, *Thermococcus onnurineus* NA1

## Abstract

The genome of the hyperthermophilic archaeon *Thermococcus onnurineus* NA1 contains three copies of the formate dehydrogenase (FDH) gene, *fdh1*, *fdh2*, and *fdh3*. Previously, we reported that *fdh2*, clustered with genes encoding the multimeric membrane-bound hydrogenase and cation/proton antiporter, was essential for formate-dependent growth with H_2_ production. However, the functionality of the other two FDH-coding genes has not yet been elucidated. Herein, we purified and characterized cytoplasmic Fdh3 to understand its functionality. The purified Fdh3 was identified to be composed of a tungsten-containing catalytic subunit (Fdh3A), an NAD(P)-binding protein (Fdh3B), and two Fe-S proteins (Fdh3G1 and Fdh3G2). Fdh3 oxidized formate with specific activities of 241.7 U/mg and 77.4 U/mg using methyl viologen and NADP^+^ as electron acceptors, respectively. While most FDHs exhibited NAD^+^-dependent formate oxidation activity, the Fdh3 of *T. onnurineus* NA1 showed a strong preference for NADP^+^ over NAD^+^ as a cofactor. The catalytic efficiency (*k*_cat_*/K*_m_) of Fdh3 for NADP^+^ was measured to be 5,281 mM^−1^ s^−1^, which is the highest among NADP-dependent FDHs known to date. Structural modeling suggested that Arg^204^ and Arg^205^ of Fdh3B may contribute to the stabilization of the 2′-phosphate of NADP(H). Fdh3 could also use ferredoxin as an electron acceptor to oxidize formate with a specific activity of 0.83 U/mg. Furthermore, Fdh3 showed CO_2_ reduction activity using reduced ferredoxin or NADPH as an electron donor with a specific activity of 0.73 U/mg and 1.0 U/mg, respectively. These results suggest a functional role of Fdh3 in disposing of reducing equivalents by mediating electron transfer between formate and NAD(P)H or ferredoxin.

## Introduction

Formate dehydrogenase (FDH), a ubiquitous enzyme in prokaryotes and eukaryotes, catalyzes the reversible oxidation of formate to carbon dioxide (CO_2_). FDHs are highly diverse in metal contents, subunit composition, types of redox cofactors, and their physiological roles (Hartmann et al., 2015). FDHs can be divided into two main classes according to their metal content/structure and catalytic strategy: metal-independent FDHs and metal-containing FDHs. The metal-containing FDH class is comprised of molybdenum or tungsten-containing enzyme families, mainly derived from bacteria and archaea, while metal-independent FDH class enzymes are more abundant in aerobic bacteria, yeasts, fungi, and plants (Maia et al., 2015). In contrast to metal-independent FDH, which mediates direct hydride transfer from formate to NAD^+^, in metal-containing FDH, the transfer of proton and electrons is mediated by the metal center in the active site and there is no direct proton/electron transfer between formate and the electron acceptor (Maia et al., 2015, 2017). The active site of FDHs is conserved and consists of three amino acids, Cys (or SeCys)-His-Arg. Many of them are known to be oxygen-labile, and FDHs containing tungsten or SeCys are generally more sensitive to oxygen (Nielsen et al., 2019). However, there are exceptions to this trend. For example, FDH of *Methylobacterium extorquens* AM1 containing tungsten was found to be oxygen tolerant (Laukel et al., 2003). The mechanism of oxygen-induced loss of activity has not yet been investigated in detail.

Prokaryotic FDHs contribute to diverse formate metabolism. For example, a hydrogen-dependent carbon dioxide reductase (HDCR) complex containing FdhF has been demonstrated to generate formate as an energy metabolic intermediate in the acetogenic bacteria *Acetobacterium woodii* and *Thermoanaerobacter kivui* (Schuchmann and Müller, 2013; Schwarz et al., 2018; Müller, 2019). Likewise, FDH in *Clostridium autoethanogenum* and *Clostridium acidurici* forms a complex with electron-bifurcating hydrogenase to reduce CO_2_ to formate as a biosynthetic precursor (Wang et al., 2013a,b). A variety of respiratory chains containing FDH benefit from formate oxidation coupled with the reduction of various terminal electron acceptors, such as nitrate, sulfate, polysulfide, fumarate, carbon dioxide, iron (Fe^3+^), arsenate, oxygen, or even protons (Kim et al., 2010; Maia et al., 2015). In *Corynebacterium glutamicum*, FdhF oxidizes formate to CO_2_ in the methanol oxidation pathway (Witthoff et al., 2013). A variety of bacteria possess a formate-hydrogen lyase composed of hydrogenase and FDH to detoxify formate as an end product during fermentation (Rittmann et al., 2015). Although the biochemical properties and physiological roles of FDHs are well studied in bacteria, research on FDHs in hyperthermophilic archaea is very limited.

On the other hand, FDHs are promising biocatalysts for the conversion of NAD(P)^+^ to NAD(P)H for industrial applications. Many industrially interesting reactions catalyzed by oxidoreductases require NAD(P)H as a cofactor, and NADPH regeneration is a key issue since most biosynthetic reactions depend on NADPH, which is not feasible to add externally (Woodyer et al., 2005; Alpdagtas and Binay, 2020). Enzymatic NADPH regeneration methods have been developed by using glucose dehydrogenase, glucose-6-phosphate dehydrogenase, alcohol dehydrogenase, and FDH. One of the weaknesses of these enzymes is the accumulation of byproducts. For example, glucose dehydrogenase produces gluconolactone, glucose-6-phosphate dehydrogenase produces 6-phosphogluconolactone, and alcohol dehydrogenase produces aldehyde as a byproduct (Eckstein et al., 2004; Lee et al., 2007; Xu et al., 2021). The accumulation of nonvolatile byproducts requires additional separation processes that can affect cost (Wang et al., 2017). FDH has advantages over other enzymes in terms of low substrate cost and no byproduct accumulation. Therefore, many researchers have attempted protein engineering using FDHs to switch their substrate specificity from NAD^+^ to NADP^+^ or to discover novel enzymes which are naturally dependent to NADP^+^ over NAD^+^ (Wu et al., 2009; Alpdagtas et al., 2018).

Previously, we reported that the hyperthermophilic archaeon *Thermococcus onnurineus* NA1 was able to grow by formate oxidation coupled with H_2_ production through the concerted activities of Fdh2 (TON_1563-TON_1564), Mfh2 hydrogenase (TON_1565-TON_1571), and Mnh2 Na^+^/H^+^ antiporter (TON_1574-TON_1580; Kim et al., 2010). Subsequently, we demonstrated that formate oxidation leads to H^+^ translocation across the cytoplasmic membrane, driving Na^+^ translocation to form a sodium motive force (Lim et al., 2014). The genome of *T. onnurineus* NA1 encodes two more copies of genes, *fdh1* (TON_0281) and *fdh3* (TON_0539), annotated as putative formate dehydrogenase catalytic subunits (Lee et al., 2008). While Fdh2 plays an essential role in formate-dependent growth, the physiological role of these FDHs has not been determined. A recent study suggested that the two gene clusters coding for a formate-hydrogen lyase and a putative formate dehydrogenase-NAD(P)H oxidoreductase may contribute to reducing equivalent disposal under specific conditions pressurized with H_2_, which is near thermodynamic equilibrium (Guellec et al., 2021). However, the suggestion related to FDHs was mostly based on bioinformatic analysis and did not accompany any characterization of FDH at the molecular level. In this study, the functional role of Fdh3 in *T. onnurineus* NA1 was investigated by purifying the enzyme and identifying its biochemical properties.

## Materials and Methods

### Strains, Medium, and Cultivation Conditions

The strains used in this study are summarized in Table 1. *T. onnurineus* NA1 strains were cultivated at 80°C in MM1 medium (Kim et al., 2013) containing 10 g yeast extract, 35 g NaCl, 0.7 g KCl, 3.9 g MgSO_4_, 0.4 g CaCl_2_∙H_2_O, 0.3 g NH_4_Cl, 0.15 g Na_2_HPO_4_, 0.03 g Na_2_SiO_3_, 0.5 g NaHCO_3_, 0.5 g cysteine-HCl supplied with 1 ml of 100X trace element solution, 20 ml of 500X Fe-EDTA solution (Holden et al., 2001), 1 ml of vitamin solution (Wolin et al., 1963), and 1 ml of 5% (w/v) Na_2_S∙9H_2_O solution per liter. For batch cultures using bioreactors, the medium was purged with argon gas (99.999%) for 1 h to maintain anaerobic conditions at 80°C as described previously (Kim et al., 2010). Then, 100% CO was continuously supplied through a microsparger at a flow rate of 50–100 ml L^−1^ min^−1^ after inoculation of cells. The agitation speed was 400 rpm, and the working volume of the bioreactors was 5 l. The pH was adjusted to 6.1–6.2 using 0.2 N NaOH dissolved in 3.5% NaCl solution.

**Table 1:** Strains and their genotype.

Strain	Parent strain	Genotype	References
*Thermococcus onnurineus* NA1			Lee et al., 2008
156T	NA1	Previously described	Lee et al., 2016
DF01	NA1	*Δfdh1* ^a^ *Δfdh2* ^b^ *Δfdh3* ^c^	This study
MF01	DF01	*P_0157_-hmg_pfu_-fdh3* ^d^	This study
MF02	156 T	*P_0157_-hmg_pfu_-fdh3A*	This study
*Escherichia coli*			
Rosetta		F- ompT*hsdSB*(rB- mB-)gal*dcm* (DE3) pRARE(CamR)	Novagen
RTN0317	Rosetta	carrying a plasmid pET-28a(+)-*fd0317*	This study

a *fdh1, TON_0266-TON_0282*.

b *fdh2, TON_1563-TON_1580*.

c *fdh3, TON_0539-TON_0542*.

d *fdh3, TON_0539-TON_0543*.

For purification of a ferredoxin (TON_0317), the *Escherichia coli* Rosetta (DE3) strain harboring the plasmid pET-28a(+)_*fd0317* was cultivated in Luria-Bertani (LB) medium. When the cell density (OD_600nm_) reached 0.6 under aerobic conditions, protein expression was induced by the addition of 1 mM isopropyl-β-D-1-thiogalactopyranoside (IPTG, Duksan, Ansan, South Korea) and incubated for 12 h under anaerobic conditions at 37°C.

### Construction of Mutants

All mutants were made by applying the gene disruption system with slight modification (Matsumi et al., 2007; Kim et al., 2015). All recombinant plasmids used in this study were constructed by the sequence and ligation-independent cloning (SLIC) method (Jeong et al., 2012). Cells were transformed and incubated in the presence of 10 μM simvastatin as a selection marker. All mutants were isolated by single colony isolation. All primers used for introduction of mutations, gene disruption, and verification of constructs are given in Supplementary Table 1.

A parental strain (Δ*fdh1* Δ*fdh2* Δ*fdh3*) was constructed by deleting three gene clusters encoding formate dehydrogenases, *fdh1* (TON_0266-TON_0282), *fdh2* (TON_1563-TON_1580), and *fdh3* (TON_0539-TON_0542). The markerless deletion mutant was generated through homologous recombination and designated DF01 (Table 1). Then, an Fdh3 overexpression mutant, designated MF01, was made as follows: the *fdh3* gene cluster (TON_0539-TON_0543) modified with a strong promoter (P*_TN0157_*) and the *hmg_pfu_* cassette were integrated into the genome of the DF01 strain between TON_1126 and TON_1127 (Lee et al., 2015). The 3-hydroxy-3-methylglutaryl coenzyme A (HMG-CoA) reductase gene from *P. furiosus* is abbreviated *hmg_pfu_*. The *fdh3* gene cluster (TON_0539-TON_0543) was amplified using the genomic DNA of *T. onnurineus* NA1 with pUC_TON0539_NHis_F and pUC_TON0543_R primers. The primer was designed to add a 6X-His tag at the N-terminus of TON_0539. The genotype of MF01 was confirmed by PCR (Supplementary Figure 1A).

The Fdh3A overexpression mutant was constructed using the 156T strain (Lee et al., 2016), in which the genomic region of the *fdh3* gene was deleted, as follows: The *fdh3A* gene (TON_0539) was amplified using the genomic DNA of *T. onnurineus* NA1 with pUCfdh3-Nhis-slic-F and pUCfdh3-Nhis-slic-R primers. The vector, modified with the addition of left and right arms for homologous recombination, was amplified with pUC-HMG-M-inv-F and pUC-HMG-M-inv-R primers and assembled with the *fdh3A* gene fragment. The *fdh3A* gene with an N-terminal 6X-His tag was integrated into the 156 T genome between TON_1126 and TON_1127, resulting in strain MF02 (Table 1; Supplementary Figure 1B).

The *E. coli* mutant with the TON_0317 gene encoding ferredoxin was constructed as follows: the TON_0317 gene was amplified using the genomic DNA of *T. onnurineus* NA1 with pET_TON0317_F and pET_TON0317_R primers and then inserted into pET-28a(+; Novagen, Madison, WI, United States). The recombinant plasmid, designated pET-28a(+)_*fd0317*, was transformed into the *E. coli* Rosetta (DE3) pLysS strain (Stratagene, La Jolla, CA, United States), resulting in strain RTN0317 (Table 1).

### Protein Purification

To purify Fdh3, strain MF01 was cultivated using a bioreactor supplied with CO gas, and the harvested cells were resuspended in buffer A (0.1 M Tris–HCl, pH 8.0, 150 mM NaCl, 19 mM KCl, and 10% glycerol) containing EDTA-free protease inhibitor cocktail (cOmplete™, Roche Diagnostics, Mannheim, Germany). Cells were disrupted by sonication and centrifuged (12,000 × *g*, 40 min, 4°C) for removal of debris. The supernatant was loaded into TALON immobilized metal affinity chromatography (IMAC) resin (Clontech, Mountain View, United States) equilibrated with buffer A. After washing with buffer B (0.1 M Tris–HCl, pH 8.0, 150 mM NaCl, 19 mM KCl, 10% glycerol, and 10 mM imidazole), the Fdh3 protein was eluted with buffer C (0.1 M Tris–HCl, pH 8.0, 150 mM NaCl, 19 mM KCl, 10% glycerol, and 300 mM imidazole). The fractions eluted from TALON IMAC were analyzed by SDS–PAGE, and the fractions containing Fdh3A were applied to a size exclusion chromatography (SEC) column, Superose 6 increase 10/300 Gl (GE Healthcare, Chicago, IL, United States) equilibrated with buffer A. Then, the SEC column was eluted with buffer A at a flow rate of 0.5 ml min^−1^, and the fractions with absorbance at 280 nm were analyzed by SDS–PAGE.

The Fdh3A protein was also purified for comparative analysis with Fdh3. Strain MF02 was cultivated using a 5-L bioreactor supplied with CO gas. Fdh3A protein was purified by one-step purification using TALON IMAC as described earlier. Ferredoxin Fd_0317_ was purified using TALON IMAC from RTN0317 cultivated under anaerobic conditions (Table 2). All purification procedures were performed in an anaerobic chamber. The concentration and purity of the protein were analyzed by protein assay dye reagent (Bio-Rad, Hercules, CA, United States) and SDS–PAGE, respectively. The purified Fd_0317_ was chemically reduced by treatment with a titanium (III)-citrate solution (Zehnder and Wuhrmann, 1976; Jones and Pickard, 1980) and oxidized by treatment with a diamide solution for 1 h at room temperature (Jung et al., 2020).

**Table 2:** Specific activities of purified Fdh3 from *Thermococcus onnurineus* NA1.

Electron donors	Electron acceptors	Specific activity (U/mg)
Formate	**MV** ^**2+**^	241.7 ± 37.9
Formate	**NAD** ^**+**^	11.2 ± 0.25
Formate	**NADP** ^**+**^	77.4 ± 3.5
Formate	**Fd**_**0317(ox)**_	0.830 ± 0.065
NADPH	**BV** ^**2+**^	108.6 ± 2.4
**NADPH**	CO_2_	1.00 ± 0.12
**Fd**_**0317(red)**_	CO_2_	0.728 ± 0.094

*One unit is equivalent to the transfer of 2 μmol of electrons per minute. Specific activity was calculated by spectrophotometric detection of the compounds marked in bold*.

### Metal Content Analysis

The metal content (W, Mo, Fe, and Se) of the protein was determined by inductively coupled plasma–mass spectrometry (ICP–MS). Approximately 2 mg of protein was analyzed by an Agilent 7,700x (Agilent Technologies, Santa Clara, United States) at the KOPTRI Institute (Seoul, South Korea).

### Biochemical Characterization of FDH

Formate-dependent methyl viologen (MV)-reducing activity was measured using 100 mM sodium formate and 2 mM MV dissolved in buffer D consisting of 0.1 M Tris–HCl (pH 8.5) and 10% glycerol and 2 mM DTT. A total of 2.4 μg of Fdh3 (or Fdh3A) was used for each reaction. The reduction of MV was monitored at 578 nm (*ε* = 9.78 mM^−1^ cm^−1^) by using a UV–VIS spectrophotometer (UV-2600, Shimadzu, Kyoto, Japan; Lovitt et al., 1988).

Formate-dependent NAD(P)^+^-reducing activity was measured using 100 mM sodium formate and 2 mM NAD(P)^+^ dissolved in buffer D. A total of 1.2 μg of Fdh3 was used for each reaction. The time-course absorbance change of NAD(P)H generation was measured at 340 nm (*ε* = 6.22 mM^−1^ cm^−1^; Elia et al., 2003). NADPH oxidation activity coupled with benzyl viologen (BV) as an electron acceptor was measured by absorbance change at 578 nm of BV.

Formate-dependent ferredoxin-reducing activity was measured using 100 mM sodium formate and 100 μM oxidized ferredoxin dissolved in buffer D. A total of 10 μg of Fdh3 was used for each reaction. The ferredoxin oxidation/reduction activity was measured based on the absorbance change at 425 nm (*ε* = 13 mM^−1^ cm^−1^ and *ε* = 28.9 mM^−1^ cm^−1^ for the reduced and oxidized forms, respectively; Aono et al., 1989; Nguyen et al., 2017).

Ferredoxin-dependent CO_2_ reducing activity was measured using 100 μM reduced ferredoxin and 10 μg of Fdh3 in buffer D saturated with N_2_:CO_2_ (8:2) gas. NADPH-dependent CO_2_ reducing activity was measured using 0.3 mM NADPH and 10 μg of Fdh3 in buffer D saturated with N_2_:CO_2_ (8:2) gas. The condition saturated with N_2_ only was used as a control.

The specific activity of Fdh3 is defined in units (U) per milligram of protein. One unit is equivalent to 2 μmol of electrons per minute. All enzymatic assays were performed in triplicate under anaerobic conditions.

The temperature and pH optima of Fdh3 were determined by measuring formate oxidation activity using MV as an electron acceptor. For the optimal temperature, the activity was measured in the range of 40–95°C in 0.1 M Tris–HCl (pH 8.5). For optimal pH, the activity was measured in 0.1 M Tris–HCl (pH 7.0–9.0) or 0.1 M potassium phosphate (pH 6.0–8.0) at 80°C.

## Western Blot Analysis

Polyclonal antibodies of Fdh2 and Fdh3 were produced after immunization of rabbits with purified proteins by Ab Frontier Co., Ltd. (Seoul, South Korea). Western blot analysis was performed as described previously (Jung et al., 2020) and analyzed using clarity western ECL substrate (Bio-Rad, Hercules, CA, United States). Chemiluminescent signals were visualized using the ChemiDoc MP imaging system (Bio-Rad, Hercules, CA, United States).

## Results

### Sequence Analysis of Fdh3

**Figure 1:**
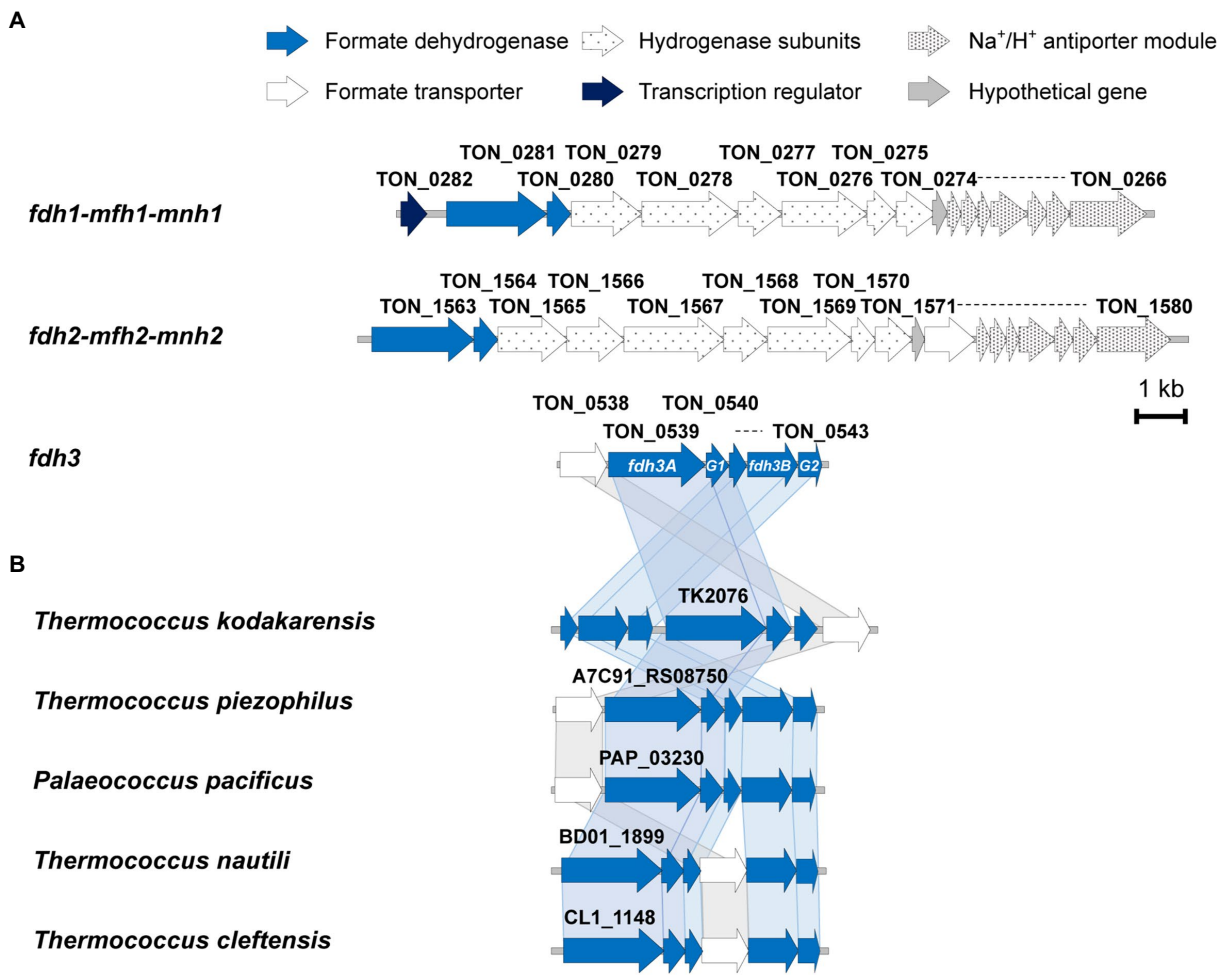
**(A)** Three formate dehydrogenase gene clusters in *Thermococcus onnurineus* NA1. The *fdh3* gene cluster is composed of six genes, from TON_0538 to TON_0543, transcribed as an operon. On the other hand, the *fdh1* and *fdh2* gene clusters are composed of three modules: a formate dehydrogenase module, a membrane-associated hydrogenase module, and a Na^+^/H^+^ antiporter module. **(B)** Conservation of the *fdh3* gene cluster in five other Thermococcales species.

The genome of *T. onnurineus* NA1 encodes three copies of genes annotated as putative formate dehydrogenase catalytic subunits, TON_0281 (*fdh1*), TON_1563 (*fdh2*), and TON_0539 (*fdh3*; Lee et al., 2008). The *fdh3* gene is clustered with genes encoding formate transporter (TON_0538), formate dehydrogenase large subunit (TON_0539), three iron–sulfur (Fe-S) proteins (TON_0540, TON_0541, and TON_0543), and NAD(P) binding protein (TON_0542; Figure 1A). Previously, it was determined that these six genes are transcribed as one transcriptional unit (Cho et al., 2017).

**Figure 2:**
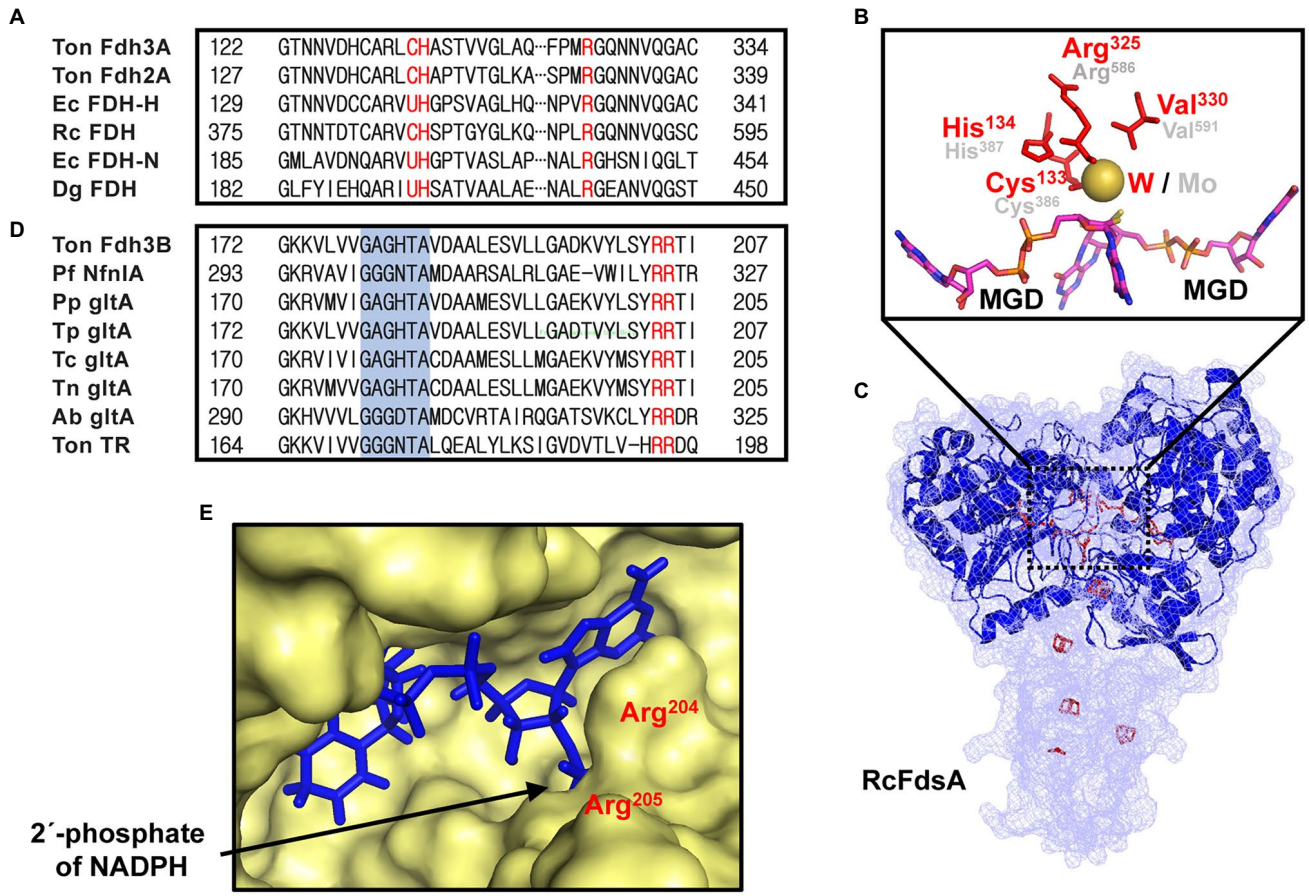
Sequence-based analysis of Fdh3A and Fdh3B. **(A)** Multiple sequence analysis (MSA) of the Fdh3A homologs with the catalytic residues Cys^133^-His^134^-Arg^325^, shown in red. **(B)** SWISS-MODEL of Fdh3A using the RcFDH cryo-EM structure as a template with the conserved residues around metal ions shown in red (Fdh3A) or gray (RcFDH). **(C)** Structure modeling of Fdh3A using SWISS-MODEL (blue ribbon) aligned with RcFdsA (mesh) (PDB-ID: 6TGA). Bis-MGD and Fe-S clusters of RcFdsA are marked in magenta and red, respectively. **(D)** MSA of homologs of Fdh3B with the FAD binding motif GXGXXG, shown in blue. **(E)** Fdh3B modeling using PfNfnI as a template with Arg^204^-Arg^205^ residues surrounding the 2′-phosphate of NADP (H) shown in red. Ton, *Thermococcus onnurineus*; Ec, *Escherichia coli*; Rc, *Rhodobacter capsulatus*; Dg, *Desulfovibrio gigas*; Pf, *Pyrococcus furiosus*; Pp, *Palaeococcus pacificus*; Tp, *Thermococcsu piezophilus*; Tc, *Thermococcus cleftensis*; Tn, *Thermococcus nautili*; and Ab, *Azospirillum brasilense*.

The gene organization of the *fdh3* gene cluster is conserved in the genomes of many Thermococcales strains (Figure 1B). Compared with the *fdh* gene clusters from strains belonging to other phyla, the *fdh3* gene clusters from Thermococcales strains appear to be unique in clustering with several genes encoding Fe-S proteins (Nielsen et al., 2019). Based on the multiple sequence alignment of various FDHs, the catalytic residues of Fdh3 of *T. onnurineus* NA1 can be pinpointed as Cys^133^-His^134^-Arg^324^, which are well conserved in *Escherichia coli*, *Rhodobacter capsulatus*, and *Desulfovibrio gigas* (Figure 2A). The structural model of TON_0539 was constructed in SWISS-MODEL^1^ using *R. capsulatus* FdsA (PDB-ID: 6TGA; Figures 2B,C),^2^ which is a subunit of the FDH complex FdsABGD, as a template (Radon et al., 2020). However, other subunits of Fdh3 of *T. onnurineus* NA1 could not be modeled using structures of *R. capsulatus* FDH or other FDHs as a template.

Motif analysis revealed that TON_0542 (*fdh3B*) was predicted to have a FAD binding motif, G-X-G-X-X-G (Figure 2D). However, the cofactor of TON_0542, a flavin-containing prosthetic group, has not been precisely identified. The structural model of TON_0542 was made in SWISS-MODEL using NADH-dependent ferredoxin NADP^+^ oxidoreductase I (NfnI) (PDB-ID: 5JCA) of *Pyrococcus furiosus* as a template. Despite substantial differences in *P. furiosus* NfnI, the FAD and NADPH binding domains of TON_0542 could be predicted (Supplementary Figure 3). Two arginine residues, Arg^204^ and Arg^205^, of TON_0542, which are known to contribute to the stabilization of the 2′-phosphate of NADP(H), are in close proximity to the NADP(H) binding pocket (Lubner et al., 2017). These Arg residues were conserved in Fdh3B homologs (Figures 2D,E).

### Subunit Composition and Metal Content

**Figure 3:**
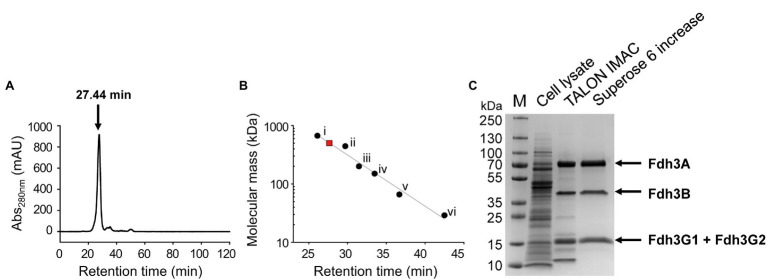
Purification of Fdh3 proteins and SDS–PAGE analysis. **(A)** Elution profile of size exclusion chromatography. **(B)** Linear regression analysis between retention time and logarithm of molecular weight of protein standards (*r*^2^ = 0.975). Protein standards were as follows: (i) thyroglobulin (669 kDa), (ii) apoferritin (443 kDa), (iii) β-amlyase (200 kDa), (iv) alcohol dehydrogenase (150 kDa), (v) albumin (66 kDa), and (vi) carbonic anhydrase (29 kDa). The Fdh3 complex corresponding to a molecular mass of 524 kDa is indicated by a red square. **(C)** SDS–PAGE of protein samples at each purification step. Ten micrograms of cell lysate and 5 μg of each of the protein samples from TALON IMAC and Superose 6 Increase were analyzed.

To purify and characterize Fdh3, an *fdh3* overexpression mutant, designated MF01, was constructed on the Δ*fdh1*, Δ*fdh2*, and Δ*fdh3* backgrounds as described in the Materials and Methods (Table 1). MF01 was cultivated in a bioreactor under CO conditions, and MF01 cell lysate was subjected to TALON immobilized metal affinity chromatography (IMAC) and size exclusion chromatography (SEC). Most of the protein eluted as a single peak in SEC, which corresponds to an estimated molecular mass of 524 kDa by the calibration curve (Figures 3A,B). Proteins that passed TALON IMAC and subsequent SEC were separated into three major bands by SDS–PAGE (Figure 3C).

These three bands were identified by MALDI mass spectrometry as TON_0539 (Fdh3A, 76.5 kDa), TON_0542 (Fdh3B, 39.1 kDa), TON_0540 (Fdh3G1, 18.3 kDa), and TON_0543 (Fdh3G2, 18.7 kDa; Supplementary Table 2). The subunit Fdh3A:Fdh3B:Fdh3G1G2 molar ratio was estimated to be 1:1:4. The exact subunit composition of Fdh3 has not yet been determined. The other two proteins of the *fdh3* gene cluster (TON_0538 and TON_0541) were not detected in the protein sample of the major fraction. The putative formate transporter (TON_0538) was predicted to be an integral membrane protein. Therefore, this protein likely interacts with other subunits of Fdh3 but was not expected to be present in the soluble fraction proteins. The absence of a putative Fe-S protein (TON_0541) in the purified protein fraction suggests that it may not be tightly bound to the complex or may not interact with other subunits of the Fdh3 complex under the conditions tested in this study.

The metal content of purified Fdh3 was determined by ICP–MS analysis. Among the tested metals, specifically, molybdenum (Mo), tungsten (W), iron (Fe) and selenium (Se), W, and Fe were detected, but Mo and Se were not. Therefore, it can be concluded that Fdh3 contains W instead of Mo as an essential metal and cysteine instead of selenocysteine as a conserved amino acid in the active site. The content of W was calculated to be less than 10% of the molar fraction of the protein, and W and Fe quantification were not evaluated stoichiometrically.

### Formate-Dependent NADP^+^ Reducing Activity of Fdh3

**Figure 4:**
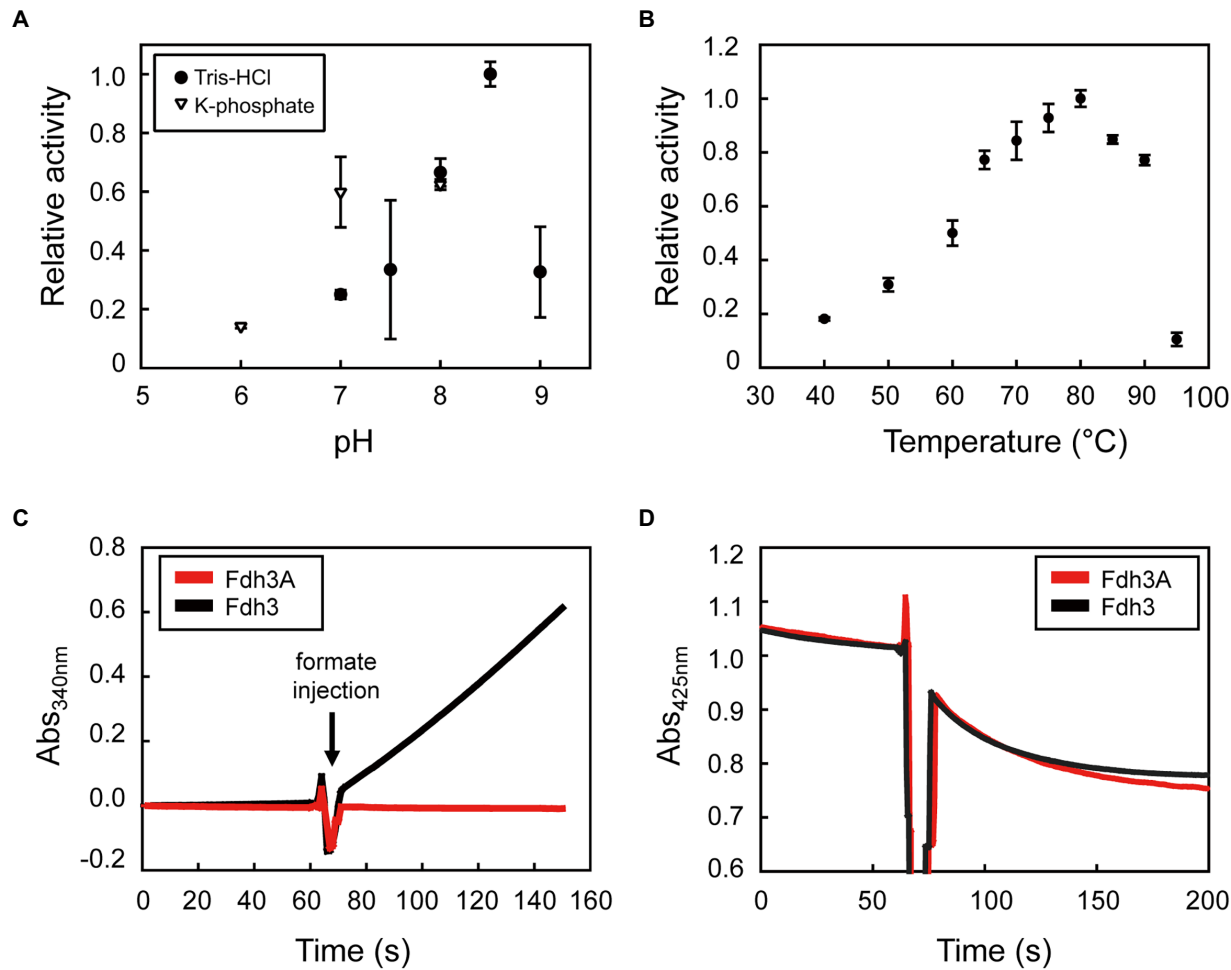
Biochemical properties of Fdh3. **(A)** The pH dependence of Fdh3 determined by formate-dependent NADP^+^-reducing activity in the pH 6.0–9.0 range in 100 mM potassium phosphate buffer (triangle) or 100 mM Tris–HCl (circle). **(B)** The temperature dependence of Fdh3 determined in the 40–95°C range in 100 mM Tris–HCl (pH 8.5) by measuring formate-dependent NADP^+^-reducing activity. **(C)** Comparison of formate-dependent NADP^+^-reducing activity between Fdh3 and Fdh3A. **(D)** Comparison of formate-dependent ferredoxin-reducing activity between Fdh3 and Fdh3A.

Based on the molecular composition of the purified Fdh3, we hypothesized that electrons formed from formate oxidation by the catalytic subunit of Fdh3A could be transferred to the NAD(P)-binding domain of Fdh3B *via* the coordination of the Fe-S clusters in Fdh3G1 and Fdh3G2. This possibility was tested using either NAD^+^ or NADP^+^, and Fdh3 displayed the activity to oxidize formate while simultaneously reducing NAD(P)^+^ to NAD(P)H (Table 2). The optimum temperature and pH conditions for the formate-dependent NADP^+-^reducing activity of Fdh3 were determined to be 80°C and pH 8.5, respectively (Figures 4A,B). The specific activity of Fdh3 to reduce NAD^+^ or NADP^+^ was determined to be 11.2 ± 0.25 U/mg and 77.4 ± 3.5 U/mg, respectively (Table 2). Fdh3A alone did not show NADP^+-^reducing activity, indicating dependence on Fdh3B (Figure 4C; Supplementary Figure 2A). The specific activities of formate-dependent methyl viologen reduction of Fdh3 and Fdh3A were similar, so the possibility of malfunction of Fdh3A due to abnormal folding or damage during purification was excluded.

The *K*_m_ and *V*_max_ of Fdh3 for formate were estimated to be 13.5 mM and 97.6 μmol/min/mg using NADP^+^ as the electron acceptor. The *K*_m_ of Fdh3 for NADP^+^ was estimated to be 0.040 mM, and the catalytic efficiency (*k*_cat_*/K*_m_) was calculated to be 5,281 mM^−1^ s^−1^, which is the highest value among NADP-dependent FDHs known to date (Table 3).

**Table 3:** Comparison of catalytic efficiency (*k*_cat_*/K*_m_) values among NADP-dependent formate dehydrogenases.

*k*_cat_*/K*_m_ value (mM^−1^ s^−1^)	Organism	References
17.9	*Mycolicibacterium vaccae*	Hoelsch et al., 2013
30	*Burkholderia stabilis*	Hatrongjit and Packdibamrung, 2010
56	*Candida methylica*	Özgün et al., 2016
5,281	*Thermococcus onnurineus* NA1	This study

### Formate-Dependent Ferredoxin-Reducing Activity of Fdh3

The possibility of Fdh3 using ferredoxin as an electron carrier was investigated. Fd_0317_ (TON_0317), one of the ferredoxins of *T. onnurineus* NA1, can be purified from *E. coli* under anaerobic conditions and tested (Supplementary Figure 2B). As a result, Fdh3 could oxidize formate by coupling with Fd_0317_ with a specific activity of 0.830 ± 0.065 U/mg (Table 2).

We tested whether Fdh3A could reduce ferredoxin. The formate-dependent ferredoxin-reducing activities of Fdh3A and Fdh3 were determined to be 16.8 ± 0.3 mU/U and 17.9 ± 1.8 mU/U, respectively, based on the same methyl viologen activity (Figure 4D). The similar activity between Fdh3A and Fdh3 means that ferredoxin does not require other subunits for interaction with Fdh3A. However, it is difficult to declare that other subunits of Fdh3 are released from Fdh3A upon binding of ferredoxins or that the ferredoxin-reducing activity is certainly dependent on only the catalytic subunit in Fdh3.

### CO_2_ Reduction Activity of Fdh3

Since most FDHs mediate the reversible reaction of formate oxidation or CO_2_ reduction, CO_2_ reduction of Fdh3 was tested with NADPH or ferredoxin as an electron donor. The CO_2_ reduction activities of Fdh3 coupled with Fd_0317_ and NADPH were determined to be 1.00 ± 0.12 U/mg and 0.728 ± 0.094 U/mg, respectively (Table 2). The formate-dependent Fd_0317_ reduction rate was similar to the Fd_0317_-dependent CO_2_ reduction rate (Table 2). However, the maximum CO_2_ reduction rate could not be determined by the limit of ferredoxin supply.

To investigate any synergistic effect of the coexistence of electron acceptors, NADPP^+^-reducing activity by formate oxidation was measured with or without Fd_0317_. The results showed that the NADPP^+^-reducing activity of Fdh3 was reduced rather than enhanced by the addition of Fd_0317_ (data not shown).

## Discussion

In this study, Fdh3 of *T. onnurineus* NA1 was purified and characterized to understand formate metabolism in the strain. The purified Fdh3 was presumed to be a heterotetrameric trimer distinct from the structure of other characterized FDHs. The subunit composition of FDH is known to be very diverse, and several FDHs with heterotetrameric structures have been reported (Hartmann et al., 2015). For example, the FDH of *Methylosinus trichosporium* OB3b is a heterotetrameric dimer, (αβγδ)_2_ (Jollie and Lipscomb, 1991). RcFDH from *R. capsulatus* was also identified as a heterotetrameric dimer, FdsABGD, where FdsD of FdsABGD was not part of FDH and was predicted to act as a chaperone for the insertion of bis-metal-binding pterin (molybdenum or tungsten) guanine dinucleotide (bis-MGD) into FdsA or a stabilizer of the quaternary structure of FdsA (Radon et al., 2020). No homolog of FdsD was detected in the *T. onnurineus* NA1 genome. Despite these examples, a heterotetrameric trimer composition is rare among FDHs. The purified Fdh3 contained only two Fe-S subunits, Fdh3G1 (TON_0540) and Fdh3G2 (TON_0543). Structure modeling of Fdh3G1 and Fdh3G2 using AlphaFold (Jumper et al., 2021) showed that the two subunits are highly similar (Supplementary Figure 4). The root-mean-square distance (RMSD) value between the two structures was calculated to be 0.608 Å. However, we cannot exclude the possibility that TON_0541 can associate with Fdh3 under certain conditions. The Fe-S cluster-rich nature of Fdh3 is interesting because Fe-S clusters may contribute to the electron relay between Fdh3A or Fdh3B and other protein(s), conferring other functional roles to Fdh3. Therefore, the study of the interaction between the Fdh3 subunits and the proteins of *T. onnurineus* NA1 expressed in various culture conditions will reveal novel enzymatic properties and *in vivo* functions. Comparative structural analysis with other FDHs can provide a spatial array of electron transfer centers in tetrameric or dimeric complexes, awaiting further study.

Purified Fdh3 could mediate NAD(P)- or ferredoxin-dependent formate oxidation and the reverse reaction. The absence of NAD(P)-dependent formate oxidation activity in Fdh3A suggests that the NAD(P)-dependent activity of Fdh3 is conserved by the catalytic subunit Fdh3A (TON_0539) and the NAD(P)^+^ binding domain of Fdh3B (TON_0542). Moreover, Fdh3G1 (TON_0540) and Fdh3G2 (TON_0543) seemed likely to transfer electrons between the two subunits. Ferredoxin-dependent activity was detected in both Fdh3A and Fdh3. The precise composition of the whole complex or spatial orientation of each subunit would help to elucidate the underlying mechanism of the biochemical properties.

*T. onnurineus* NA1 Fdh3 showed high *k*_cat_/*K_m_* value toward NADP^+^, which can be attributed to the high optimum temperature and low *K_m_* value toward NADP^+^. Thermophilic FDH of *Moorella thermoacetica* has been reported and the optimum temperature was 70° and 80° for the electron acceptors NADP and MV, respectively (Andreesen and Ljungdahl 1974; Ljungdahl and Andreesen, 1978). The specific activity of formate oxidation using NADP as an electron acceptor was determined to be 34 U/mg for *M. thermoacetica* FDH and 77.4 U/mg for *T. onnurineus* NA1 Fdh3. *k*_cat_/*K_m_* value toward NADP^+^ has not been determined for *M. thermoacetica* FDH. However, the specific mechanism of cofactor dependence and catalytic efficiency are still veiled. The *K_m_* value of Fdh3 toward formate appeared high (13.5 mM). High *K_m_* values toward formate have also been reported for other FDHs, such as *Moraxella* sp. strain C-1 (13 mM; Asano et al., 1988), *Komagataella pastoris* (15 mM; Allais et al., 1983), *Bacillus* sp. F1 (19.6 mM; Ding et al., 2011) and *Kloechkera* sp. No. 2201 (22 mM; Kato et al., 1974).

From a biotechnological perspective, the high conversion efficiency of Fdh3 toward NADPH offers an option for enzymatic NADPH regeneration. Glucose dehydrogenase (Weckbecker and Hummel, 2005), glucose-6-phosphate dehydrogenase (Lee et al., 2007), and alcohol dehydrogenase (Xu et al., 2021) are known to have significantly high conversion efficiency toward NADPH. Unlike the above mentioned enzymes, FDH is advantageous in that it produces only CO_2_ without the accumulation of other byproducts. According to the structure modeling of Fdh3B, Arg^204^-Arg^205^ residues in the putative NADPH binding pocket play an important role to stabilize the 2’-phosphate of NADP(H).

Fdh3 of *T. onnurineus* NA1 was shown to use ferredoxin as an electron carrier. Based on the Fdh3A data, the other subunits, Fdh3B, Fdh3G1, and Fdh3G2, did not seem essential for ferredoxin-dependent activity. The interaction between ferredoxin and FDH has been reported in *Thermococcus kodakarensis* (Burkhart et al., 2019). Fd-2 ferredoxin (TK1087) and FdhA (TK2076) were identified to interact by interactome analysis (Burkhart et al., 2019). Fd-2 and FdhA of *T. kodakarensis* share 88 and 57% identities with Fd_0317_ and Fdh3A of *T. onnurineus* NA1, respectively. We also tested formate oxidation or CO_2_ reduction by Fdh3 using another ferredoxin, Fd_1361_, of *T. onnurineus* NA1 and found highly unstable activity (data not shown). Fd_1361_ shares 73% identity with another Fd-1 ferredoxin (TK1694) of *T. kodakarensis*, of which no interaction with FdhA was observed in the interaction data (Burkhart et al., 2019).

Although ferredoxin-reducing activity was detected, it is not clear whether ferredoxin-dependent formate oxidation can occur *in vivo*. Since most ferredoxins are known to be in a reduced state under anaerobic biological conditions, assuming that the redox potential of ferredoxin is as low as −500 mV, ferredoxin-dependent CO_2_ reduction is energetically more favorable than the opposite direction (Li and Elliott, 2016). The first reported ferredoxin-dependent FDH, also called CO_2_ reductase, is from *Clostridium pasteurianum* (Scherer and Thauer, 1978). FDH consists of two subunits, FdhA (76 kDa) and FdhB (34 kDa), and was identified to reduce CO_2_ to formate with reduced ferredoxin and oxidize formate using ferredoxin as an electron acceptor (Liu and Mortenson, 1984). Recently, it has been reported that ferredoxin reduced by carbon monoxide dehydrogenase can mediate CO_2_ reduction by HDCR (Schuchmann and Müller, 2013). In addition to the purified enzyme assay, CO_2_ reduction by HDCR using ferredoxin was also implicated in the resting cell assay in the wild-type strain and ∆*hydBA* and ∆*rnf* mutant strains of *A. woodii* (Schwarz et al., 2020). Comparative structural analysis between enzymes may provide a spatial array to elucidate the electron relay mechanism, which awaits further study.

**Figure 5:**
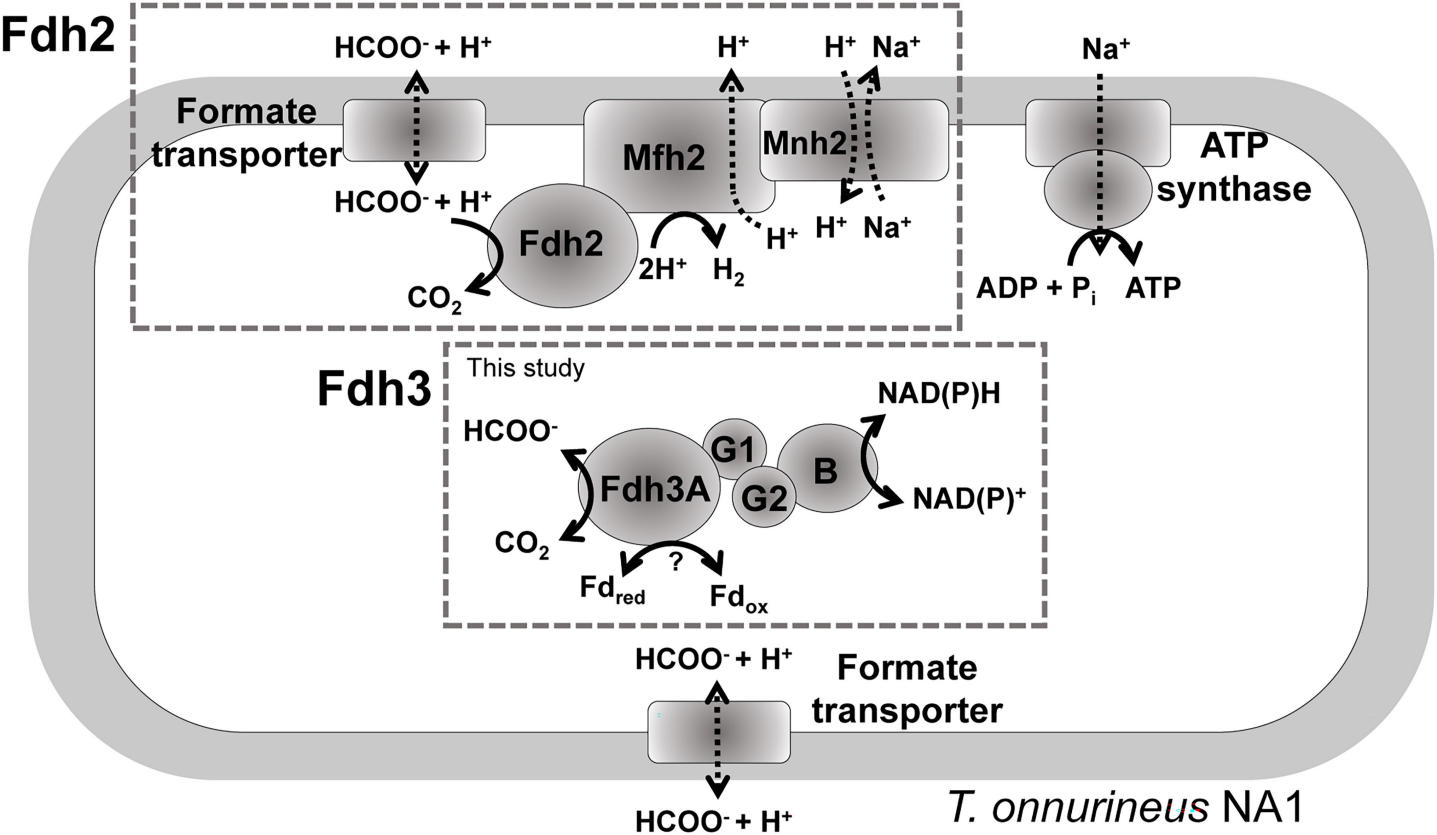
A proposed model for the functional roles of Fdh2 and Fdh3. The schematic diagram does not represent the actual composition of each subunit of Fdh3.

A recent study suggested that gene clusters encoding formate hydrogenlyase or putative FDH-NAD(P)H oxidoreductase may contribute to reducing equivalent disposal under certain conditions with hydrogen pressure (Guellec et al., 2021). Previously, we showed that the *fdh2* gene cluster composed of Fdh2 (TON_1563-TON_1564), Mfh2 hydrogenase (TON_1565-TON_1571), and Mnh2 Na^+^/H^+^ antiporter (TON_1574-TON_1580) is essential for formate-dependent growth with a series of knockout experiments. Furthermore, the generation of osmotic electron potential and ATP was clearly demonstrated using resting cells in *T. onnurineus* NA1, experimentally demonstrating the role of Fdh2 in ATP generation (Kim et al., 2010; Lim et al., 2014). Even though the *fdh2* gene cluster is mainly responsible for formate oxidation coupled with hydrogen production, the catalytic subunit of Fdh2 may also mediate CO_2_ reduction as well as formate oxidation under certain conditions, partly because most formate dehydrogenases mediate reversible reactions of formate oxidation or CO_2_ reduction. This study demonstrated that reducing equivalents such as NAD(P)H and ferredoxin could be disposed of by Fdh3 (Figure 5). The contribution of Fdh3B (TON_0542) with NAD(P)^+^ binding domain appears to be important in NAD(P)H disposal. The possibility of Fdh3B working with Fdh2 can be ruled out in two respects. First, the gene encoding Fdh3B was transcribed into a single operon with the fdh3 gene cluster (Cho et al., 2017), whose gene products form a stable complex. Second, we previously reported that the expression of the Fdh3 gene cluster was distinctive from that of the Fdh2 gene cluster in *T. onnurineus* NA1. Fdh3 was upregulated under sulfur-containing conditions, whereas Fdh2 was highly upregulated in the presence of CO or formate (Cho et al., 2017). Meanwhile, Western blotting analysis showed that Fdh3 and Fdh2 were expressed under different conditions (Supplementary Figure 5). At the protein level, Fdh3 was highly expressed when carbohydrates or CO was added and weakly expressed under sulfur-containing conditions. On the other hand, Fdh2 was highly expressed in pyruvate-containing medium followed by maltodextrin, formate and CO conditions.

Conclusively, purification and characterization of Fdh3 provided information on the functionality of the protein, suggesting a distinct role for FDH in hyperthermophilic archaea. Many questions remain, and further research will answer them.

## Data Availability Statement

The original contributions presented in the study are included in the article/Supplementary Material, further inquiries can be directed to the corresponding authors.

## Author Contributions

SK and HL designed the research. SL and J-YR carried out the experiments. J-iY interpreted the bioinformatic and experimental data analyses. J-iY, SK, and HL wrote the manuscript. All authors have read and approved the manuscript.

## Funding

This study was funded by the KIOST In-House Program (grant number PE99922) and the Development of Biohydrogen Plant Operation Optimization System program of the Ministry of Oceans and Fisheries in the South Korea.

## Conflict of Interest

The authors declare that the research was conducted in the absence of any commercial or financial relationships that could be construed as a potential conflict of interest.

## Publisher’s Note

All claims expressed in this article are solely those of the authors and do not necessarily represent those of their affiliated organizations, or those of the publisher, the editors and the reviewers. Any product that may be evaluated in this article, or claim that may be made by its manufacturer, is not guaranteed or endorsed by the publisher.

## Footnotes

^1^ http://swissmodel.expasy.org/

^2^ https://www.rcsb.org/
