# Supplementary material for: A Novel NADP-Dependent Formate Dehydrogenase From the Hyperthermophilic Archaeon Thermococcus onnurineus NA1
Source: Front Microbiol. 2022 Mar 15;13:844735. doi: 10.3389/fmicb.2022.844735 (PMC8965080; doi:10.3389/fmicb.2022.844735)
Supplement: Supplementary file 1 [file Data_Sheet_1.docx]

***Supplementary Material***

**Supplementary Table 1**. Primers used in this study.

| Mutant name | Primer name | Sequence (5' → 3') |
| --- | --- | --- |
| DF01 | fdh1_LA_F | attcgagctcggtacccggatataacgccaatgacctcg |
|  | fdh1_LA_R | cagtgccaagcttgcatgcggatcccaccaacccgtgatgactga |
|  | fdh1_RA_F | tcagtcatcacgggttggtgatctaagagcatggcattcg |
|  | fdh1_RA_R | cagtgccaagcttgcatgcactccagcgcggaacgcaga |
|  | fdh2_LA_F | attcgagctcggtacccggaagagtttctcgccgaacttc |
|  | fdh2_LA_R | cagtgccaagcttgcatgcggatcctctccacacctccacattgg |
|  | fdh2_RA_F | ccaatgtggaggtgtggagatactggcagtcatctgcctg |
|  | fdh2_RA_R | cagtgccaagcttgcatgcggtttaggaagcggctagcc |
|  | fdh3_LA_F | aattcgagctcggtacccggggatccatggcacagaataattcactcg |
|  | fdh3_LA_R | ggccatccttaacagcaccaccgccctatcttc |
|  | fdh3_RA_F | gatagggcggtggtgctgttaaggatggcctgtatg |
|  | fdh3_RA_R | ccagtgccaagcttgcatgcctgcaggaatgaccttggttatgccg |
| MF01 | pUC_TON0539_NHis_F | gggctcataaaatgcttgggagatgacctaggatgcaccatcaccaccatcacgaggagtttaagattggcctgtgc |
|  | pUC_TON0543_R | ggctggccatcgttgacgccaccctaggttaaagcatcctcccaacacc |
| MF02 | pUCfdh3-Nhis-slic-F | gggagatgacctaggatgcaccatcaccaccatcacgaggagttta |
|  | pUCfdh3-Nhis-slic-R | tgacgccaccctaggtcagcaccccccaat |
|  | pUC-HMG-M-inv-F | cctagggtggcgtcaacgat |
|  | pUC-HMG-M-inv-R | cctaggtcatctcccaagca |
| RTN0317 | pET_TON0317_F | tgccgcgcggcagccatatgccggagaagattaaagtcgtc |
|  | pET_TON0317_R | tggtggtggtggtgctcgagttcggacacccccaggg |

**Supplementary Table 2**. Identification of Fdh3 using LC–MS/MS analysis.

| Protein identified | Accession  number | Locus tag | Unique peptides  matched | Sequence  coverage | Mascot Protein score |
| --- | --- | --- | --- | --- | --- |
| Hypothetical formate dehydrogenase, α subunit | ACJ16026 | TON_0539 | 247 | 58% | 1825 |
| Glutamate synthase ß chain-related oxidoreductase | ACJ16029 | TON_0542 | 570 | 53% | 1386 |
| Oxidoreductase iron-sulfur protein | ACJ16027 | TON_0540 | 105 | 92% | 1338 |
| 4Fe-4S cluster-binding protein | ACJ16030 | TON_0543 | 100 | 92% | 1266 |


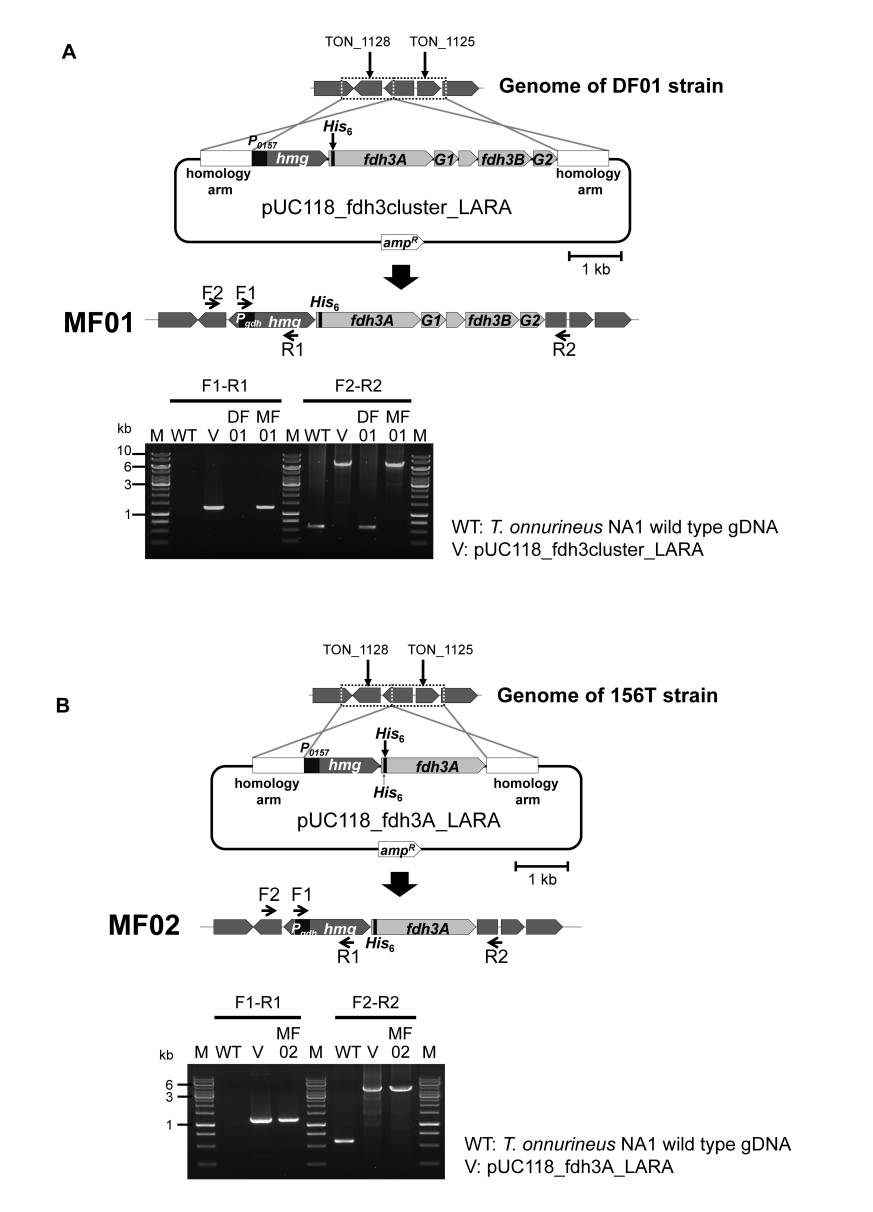


**Supplementary Figure 1**. Schematic diagram of the genotype of *T. onnurineus* NA1 mutants used in this research. **(A)** Genotype confirmation of DF01 and MF01. **(B)** Genotype confirmation of MF02. *P_0157_*, promoter of glutamate dehydrogenase gene (TON_0157); *hmg*, HMG-CoA reductase gene (PF1848) from *P. furiosus*.


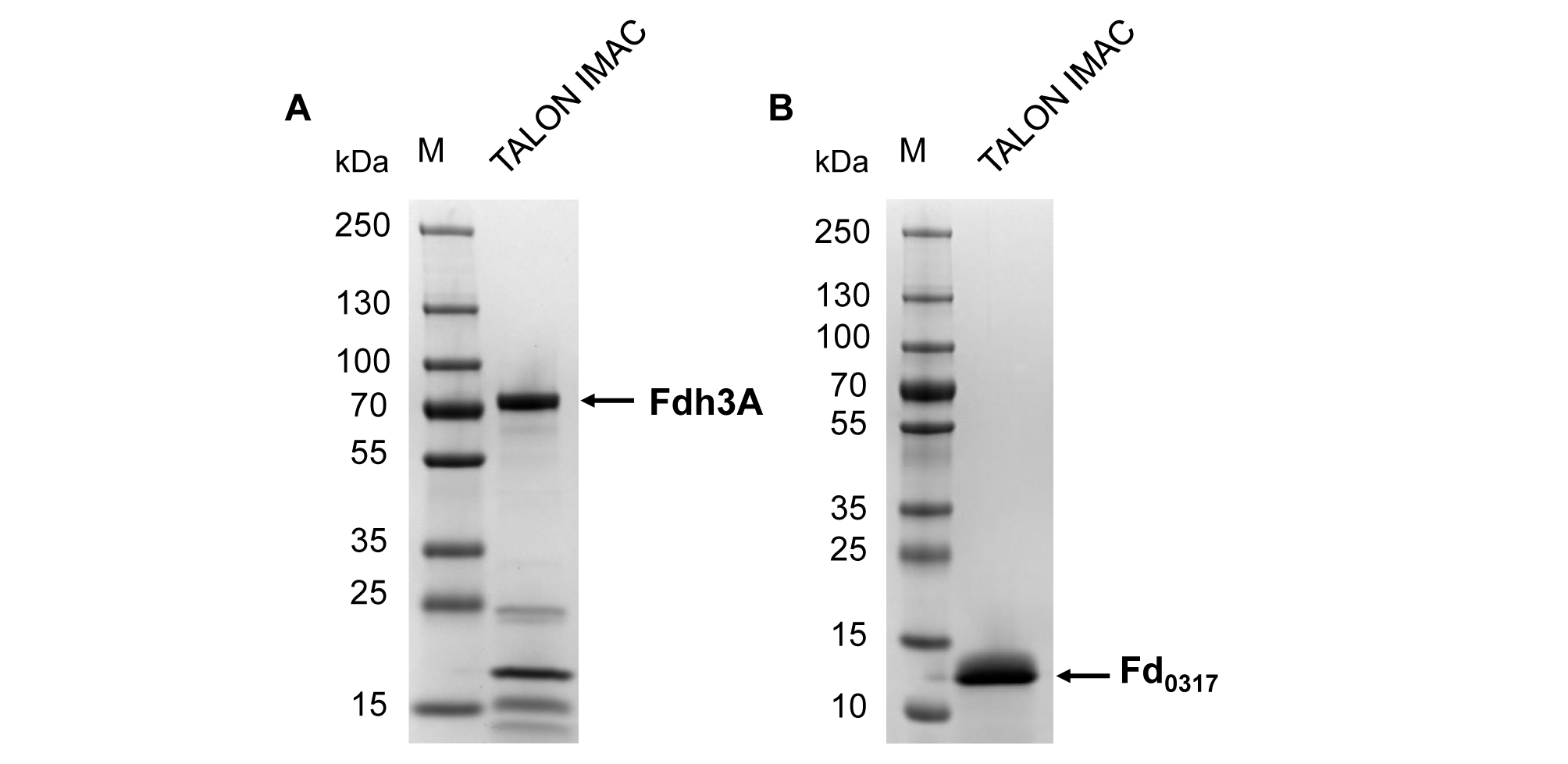


**Supplementary Figure 2**. Purification and SDS–PAGE analysis of Fdh3A **(A)** and Fd_0317_ **(B)** proteins purified from TALON IMAC.


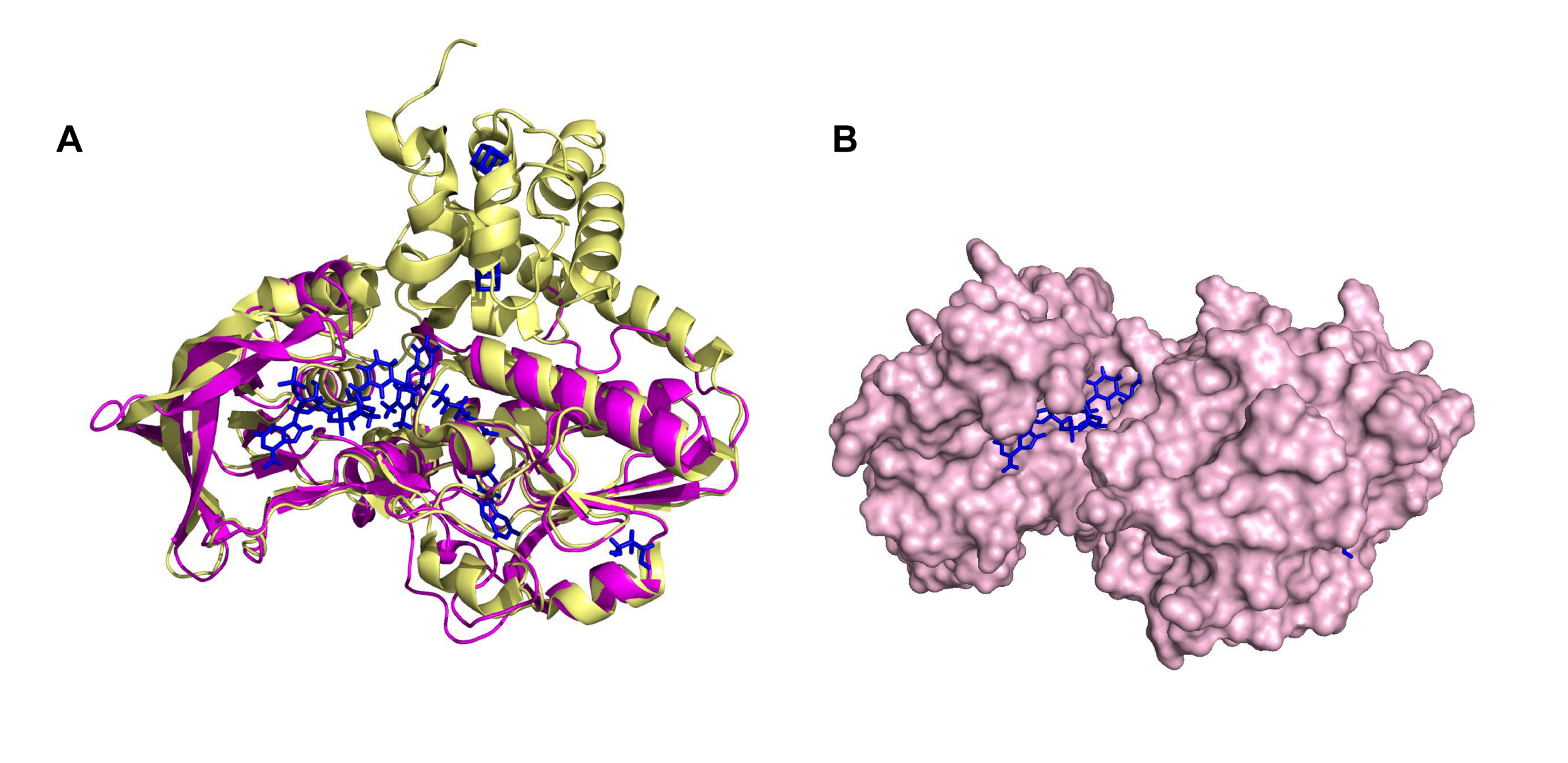


**Supplementary Figure 3**. **(A)** Structure modeling of Fdh3B (magenta) using SWISS-MODEL superimposed with PfNfnI-L (yellow, PDB-ID: 5JCA). FAD, NADPH and Fe-S clusters are marked in blue. **(B)** FAD (blue) embedded in binding pocket of Fdh3B (pale pink).


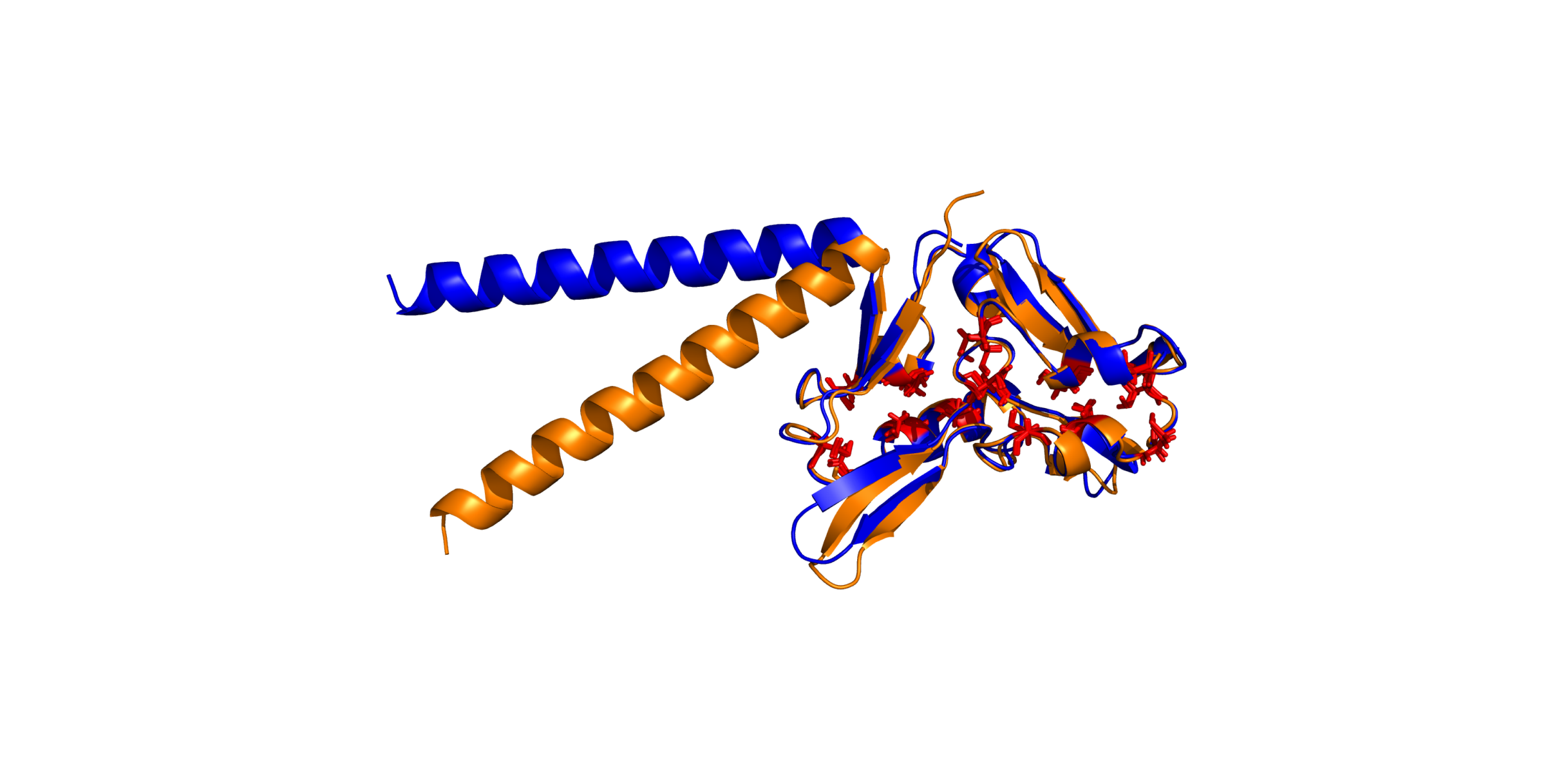


**Supplementary Figure 4**. Structure modeling of Fdh3G1 and Fdh3G2 using AlphaFold. Fdh3G1 in blue; Fdh3G2 in orange; cysteine residues in red.


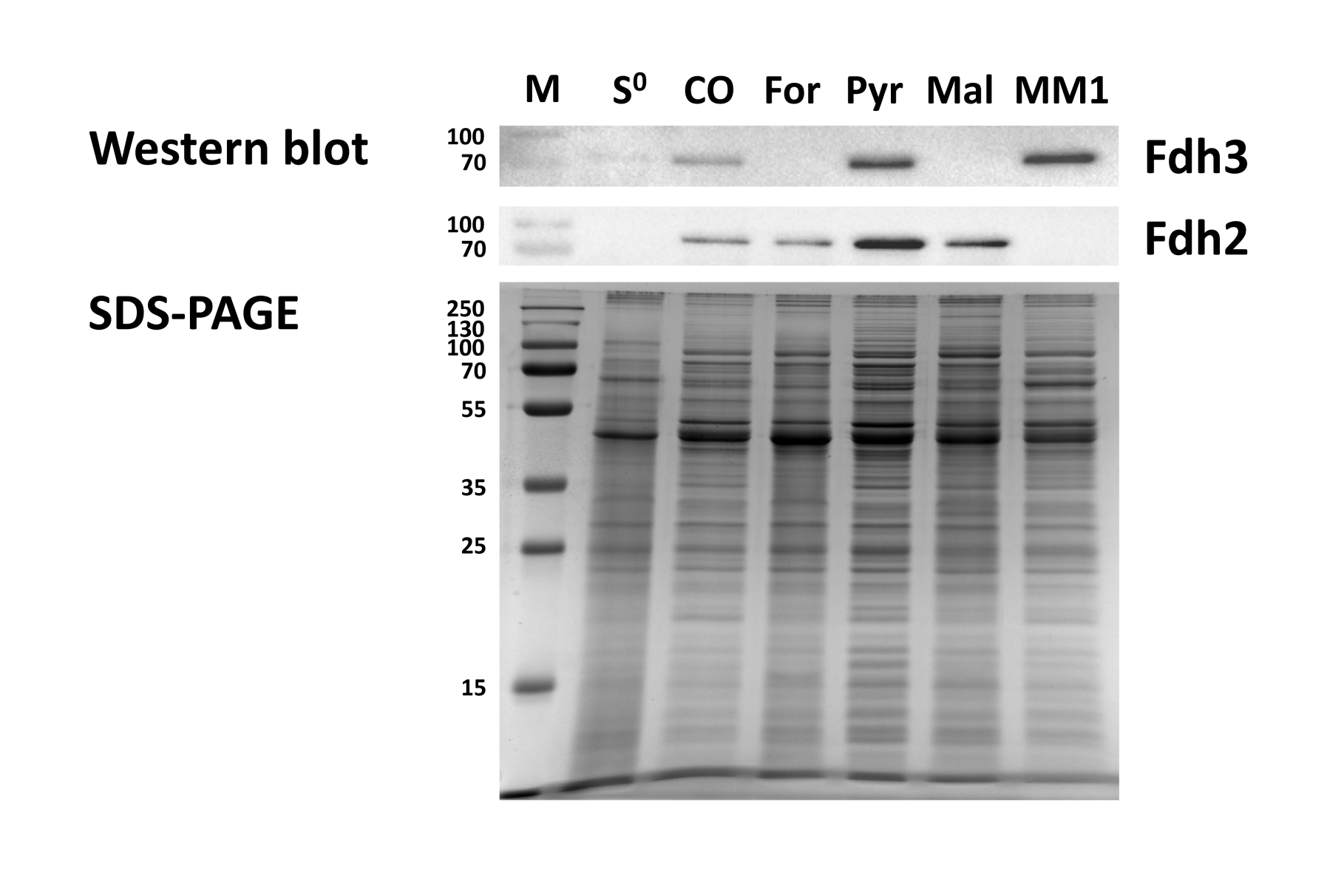


**Supplementary Figure 5**. Western blotting of Fdh2 and Fdh3 under various conditions. S^0^, ASW-YT containing elemental sulfur; CO, MM1 charged with CO; For, MM1 containing sodium formate; Pyr, MM1 containing sodium pyruvate; Mal, MM1 containing maltodextrin.
